# Triple Threat: Significant Concomitant Aortic Stenosis, Mitral Stenosis, and Systolic Anterior Motion of the Mitral Valve Causing Left Ventricular Outflow Tract Obstruction in Cardiac Surgical Patients

**DOI:** 10.1155/2023/9995115

**Published:** 2023-03-17

**Authors:** Katelyn Glines, J. W. Awori Hayanga, Christina Gibson, Mohamad El Churafa, Lawrence Wei, Heather K. Hayanga

**Affiliations:** ^1^West Virginia University Hospitals, Department of Anesthesiology, Medical Center Drive, Morgantown 26505, WV, USA; ^2^West Virginia University Hospitals, Department of Cardiovascular and Thoracic Surgery, Medical Center Drive, Morgantown 26505, WV, USA

## Abstract

Systolic anterior motion (SAM) describes a pathologic condition of the mitral valve in which the anterior leaflet is displaced anteriorly, resulting in a narrowed left ventricular outflow tract (LVOT). The implications of SAM may range in severity from clinically insignificant disease to severe LVOT obstruction resulting in hemodynamic collapse. While SAM is typically observed in patients with hypertrophic cardiomyopathy or following mitral valve repair, it may be seen in any setting in which the anatomy and function of the left ventricle has been altered. Here we discuss two patients who presented for aortic and mitral valve replacements for concomitant aortic and mitral stenosis. These cases were further complicated by the preoperative diagnosis of SAM in addition to the preexisting valvular lesions, further increasing the risk of sudden hemodynamic collapse and cardiac arrest.

## 1. Introduction

A variety of underlying conditions may result in the development of systolic anterior motion (SAM) of the mitral valve (MV), with two main proposed mechanisms: Venturi and drag forces. In the Venturi mechanism, the rapid velocities in the left ventricular outflow tract (LVOT) lift the mitral valve toward the septum. Drag forces move the leaflet anteriorly, increasing the outflow tract gradient as the leaflet moves toward the septum causing an obstruction [1].

Patients with or without hypertrophic cardiomyopathy (HCM) may present with SAM. The prevalence of SAM in HCM is approximately 30–60%, with LVOT obstruction demonstrated in 25–50% of these cases [2]. Additionally, the incidence of SAM is thought to be 1–16% after surgical repair of the mitral valve [3].

The patients discussed in this report present in a unique context: the coexisting presence of significant native valve aortic stenosis (AS), mitral stenosis (MS), and SAM. The anesthetic management of these patients necessitated a heightened understanding of the anatomic basis, structural function, and potential hemodynamic consequences of the associated pathologies. Transesophageal echocardiography was an invaluable tool in classification of disease severity, anesthetic management, and providing surgical guidance.

## 2. Case Presentation

### 2.1. Patient 1

**Figure 1:**
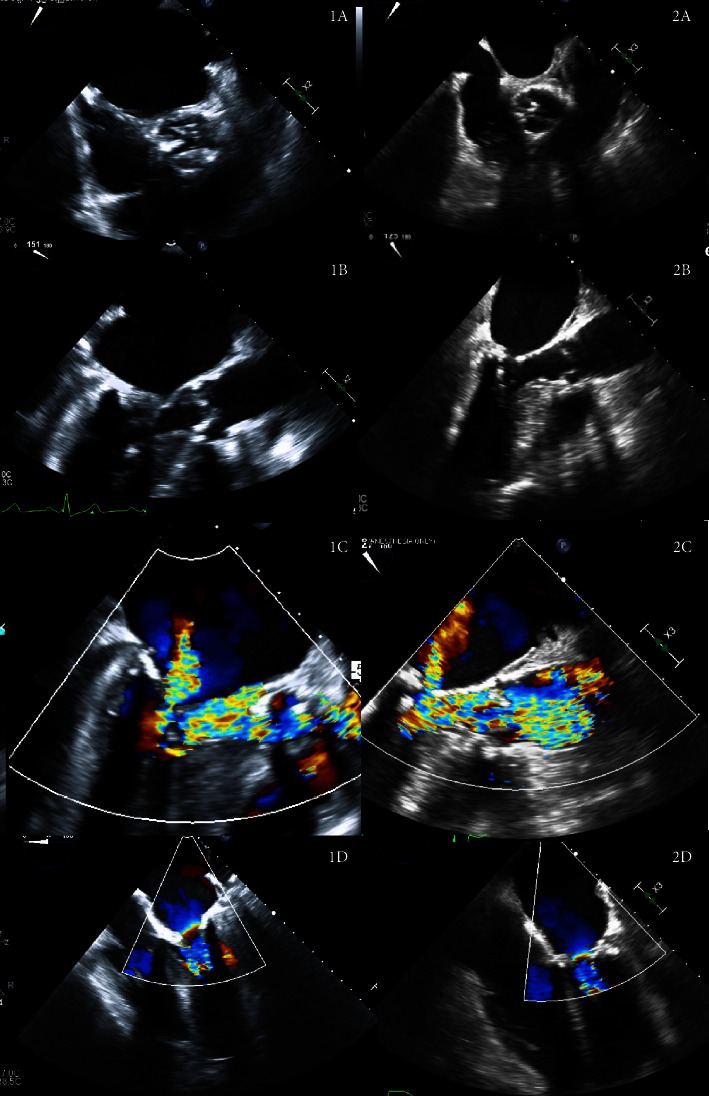
Pre-cardiopulmonary bypass transesophageal echocardiographic views for Patient 1 (1A–1D) and Patient 2 (2A–2D). A: midesophageal aortic valve short-axis view. B: midesophageal long-axis view during systole with SAM. C: midesophageal long-axis view during systole with SAM and color-flow Doppler. D: midesophageal four-chamber view with color-flow Doppler.

A 72-year-old female with severe AS (mean gradient of 47 mm·Hg and aortic valve area of 0.4 cm^2^), moderate MS (mean gradient of 6 mm·Hg), moderate mitral regurgitation, and HCM presented for aortic and mitral valve replacement as well as septal myectomy. Preoperative echocardiography showed severe left ventricular hypertrophy with a preserved ejection fraction of 65%. With standard monitoring after invasive arterial line placement and a 500 mL crystalloid bolus, induction of anesthesia was achieved with 500 mcg fentanyl and 2 mg intravenous midazolam in small, divided doses. Boluses of phenylephrine in doses of 50–100 micrograms (mcg) were used as needed to maintain a mean arterial pressure (MAP) near baseline of 80–95 mm·Hg. The majority of phenylephrine boluses were administered during the time between induction and incision and immediately prior to retrograde autologous priming (RAP) of the cardiopulmonary bypass (CPB) circuit. Pre-cardiopulmonary bypass transesophageal echocardiography (TEE) was performed confirming the aortic and mitral valvular pathologies and also demonstrating SAM causing LVOT obstruction (1A–1D in Figure 1). The surgical intervention was of increased complexity due to severe mitral annular calcification and presence of septal hypertrophy. The patient underwent aortic and mitral valve replacements with a 21 mm Medtronic Avalus™ bioprosthetic valve and 27 mm Abbott Epic^TM^ stented tissue valve, respectively. Septal myectomy was also performed. Mean gradient across the bioprosthetic aortic and mitral valves was measured to be 11 mm·Hg and 3 mm·Hg, respectively, on post-cardiopulmonary bypass TEE. No paravalvular leaks or central regurgitant jets were evident. The left ventricular outflow tract showed laminar flow using color-flow Doppler without evidence of a ventricular septal defect. The postoperative course was complicated by need for a permanent pacemaker on postoperative day 5 for ventricular standstill. On postoperative day 7, the patient experienced a rapid decline in clinical status. While receiving one unit of packed red blood cells, she developed nausea, chills, hypotension, and hypoxia. Transfusion reaction was suspected. However, confirmatory workup was later negative. Following transfer to the intensive care unit, her pacemaker was interrogated. She was found to be in new-onset atrial fibrillation with adequate rate control in the 80s. Minutes later she had seizure-like activity and became unresponsive, hypotensive, and hypoxic. Emergent intubation was performed. Immediately following intubation and initiation of mechanical ventilation, she became asystolic. Cardiopulmonary resuscitation and bedside cardiac massage were performed with no return of spontaneous circulation.

### 2.2. Patient 2

In a similar case, a 71-year-old female with moderate AS (mean gradient of 30 mm·Hg and aortic valve area of 1.0 cm^2^), severe MS (mean gradient of 15 mm·Hg), and mild mitral regurgitation presented for aortic and mitral valve replacement. The left ventricle was noted to be small and hyperdynamic. Preoperative cardiac catheterization showed 50% proximal and 80% mid-stenosis of the left anterior descending artery. In addition to the valvular lesions, preoperative echocardiography was notable for a left ventricular ejection fraction of 65%, significant mitral annular and leaflet calcification, and chordal SAM with LVOT obstruction without septal hypertrophy (2A–2D in Figure 1). With standard monitoring and after invasive arterial line placement and a 500 mL crystalloid bolus, induction of anesthesia was achieved with 350 mcg fentanyl, 30 mg ketamine, and 3 mg intravenous midazolam in small, divided doses. Phenylephrine boluses were given in doses of 100–200 mcg as needed to maintain a MAP near the patient's baseline of 70–80 mm·Hg. A phenylephrine infusion was initiated on induction and was titrated ranging from 0.2–0.8 micrograms/kilogram/minute. As with the previous patient, phenylephrine requirements were highest from induction to incision and as hemodynamic support during RAP. Surgical complexity was increased by presence of SAM and moderate mitral annular calcification. The patient underwent CryoLife On-X®Conform-X Size 25/33 mechanical mitral valve replacement and CryoLife On-X®Conform-X 21 mm mechanical aortic valve replacement. She also underwent coronary artery bypass grafting of the left internal mammary artery to the left anterior descending artery. Post-cardiopulmonary bypass TEE showed a mean gradient of 9 mm·Hg across the aortic valve and 2 mm·Hg across the mitral valve. No paravalvular leaks or central regurgitant jets were evident. The postoperative course was complicated by subtherapeutic anticoagulation with warfarin initiation, which prolonged length of stay. The patient was discharged home on postoperative day 11.

## 3. Discussion

Few reports exist discussing the combination of significant AS and SAM, MS and SAM, or concomitant AS and MS [4, 5]. Literature describing the combination of all three lesions is even scarcer. These lesions may be very poorly tolerated in solidarity and, when combined, may conceivably increase the risk of cardiac death as well as severe hemodynamic perturbations intraoperatively. A review of the Society of Thoracic Surgeons dataset of 623,039 patients found the operative mortality rate to be nearly double in multivalvular disease when compared to single-valve procedures [6]. Approach to these patients, thus, requires a thorough preoperative evaluation, understanding of pathophysiological hemodynamic consequences, and careful anesthetic planning. It is important to be aware of the physiological interplay between MS, AS, and indicated surgical interventions. In the presence of concomitant mitral and aortic stenosis, the clinical features of mitral stenosis may predominate. The decrease in left ventricular filling results in a reduction of cardiac output, pressure gradient across the aortic valve, and ventricular stress [7]. The masking of aortic stenosis by the coexisting mitral stenosis may lead to underestimating or misclassifying the degree of aortic stenosis. Studies have shown that in the setting of mitral and aortic stenosis, replacement of only the mitral valve leads to a marginal increase in aortic valve area (AVA) by continuity equation but also a simultaneous increase in peak pressure gradients across the aortic valve [8]. Thus, it is critical to note the surgical implications of replacing the aortic valve at a later date vs. simultaneously with the mitral valve, as the severity of aortic stenosis may either be underestimated due to low gradient or overestimated due to low flow.

Multiple factors may lead to the development of SAM, including both anatomic and kinetic changes. Structural changes of the left ventricle such as acquired hypertrophy, elongation of the MV leaflets, displacement of the papillary muscles, chordal anomalies, and a small, hyperdynamic left ventricle may predispose to SAM. These structural changes may then alter the kinetics of the ventricle, namely, via Venturi and drag mechanisms, which may lead to the development of, or worsening of preexisting, SAM [3, 9]. The combination of both these mechanisms is thought to cause SAM. Drag force is a key contributor to the development of an LVOT gradient. During systole, ejection flow drag forces push the mitral valve leaflets toward the septum. The flow through the narrowed LVOT creates Venturi forces that maintain, or increase, the obstruction by lifting the leaflets toward the septum. While both contribute to LVOT obstruction, most studies suggest drag forces as the dominant cause of SAM [1, 10, 11]. A comparison of these concepts is illustrated in Table 1.

Echocardiography and cardiac magnetic resonance imaging are valuable preoperative tools in the evaluation and classification of SAM. Use of TEE is essential due to its ability to capture dynamic anatomical changes and to guide mitral valve repair strategies for those at increased risk of SAM. Statistically significant predictors of SAM include the distance of mitral valve leaflet coaptation point to the septum (C-sept) of less than 2.5 cm, anterior to posterior leaflet length less than or equal to 1.3, posterior leaflet length greater than 1.5 cm, end-diastolic left ventricular diameter less than 45 mm, left ventricular basal septal thickness greater than 15 mm, and an aorto-mitral angle less than 120 degrees [12]. Mitral annular dimensions, features of the intraventricular septum, and LVOT size and gradient should also be evaluated [13].

Fortunately, many hemodynamic goals in the anesthetic management of aortic and mitral stenosis are similar: sustain preload and afterload, maintain normal sinus rhythm, avoid tachycardia, and maintain myocardial contractility [14]. It is critical to have control of sympathetic output in the intraoperative setting from tracheal intubation to surgical stimulation with beta blockers and/or opioids to maximize left ventricular filling time and prevent elevation of trans-valvular pressure across the mitral valve which can subsequently cause a rise in left atrial and pulmonary artery pressures [15]. The use of alpha agonists with a balanced anesthetic to maintain afterload will ensure adequate coronary perfusion and augment forward flow. Meanwhile, avoidance of possible arrhythmias and maintaining normal sinus rhythm are essential. The preservation of an atrial kick contributes up to 40% of cardiac output in the setting of stenotic valves [15].

Similarly, hemodynamic goals in SAM include increased preload, afterload, and avoidance of tachycardia (Table 2). In SAM, however, decreased myocardial contractility is favorable since an increased inotropic state may decrease MV leaflet-septal distance and provoke SAM [13, 14]. The degree of LVOT obstruction seen in SAM may thus be exacerbated by decreased preload or afterload and increased contractility due to its dynamic nature [14]. In accordance with these goals, fentanyl and midazolam were the selected primary induction medications in our patients. A balanced induction technique of narcotics and benzodiazepines is favorable due to their minimal myocardial depressant properties. Narcotics blunt the sympathetic response to intubation, preventing undesirable tachycardia and hypertension that would increase myocardial oxygen demand. Additionally, the use of opioids decreases the stress response to surgical stimulation. While narcotics maintain cardiovascular stability, they lack amnestic properties. The addition of benzodiazepines provides amnesia and additive central nervous system depression [16]. Phenylephrine was the vasopressor of choice to support hemodynamics in our two patients in order to maintain afterload and aortic end-diastolic pressure, thereby maintaining coronary perfusion pressure. Reflexive bradycardia associated with phenylephrine use was also desirable. To prevent sudden hemodynamic collapse, the anesthetic management of our patients required careful monitoring and precise medication adjustments, especially during pivotal points in the case with potential for rapid hemodynamic changes.

Definitive treatment of significant mitral or aortic stenosis involves valve replacement with a prosthetic valve. Definitive treatment of SAM, however, depends on the severity of the disease, with surgical management being dependent on the specific lesion [3]. Isolated SAM, in the absence of clinical severity, is not an indication for surgery. Additionally, medical management may be sufficient in the management of mild disease [3]. When surgery is deemed necessary, some techniques target the mitral leaflet coaptation point including edge-to-edge repairs [3]. Other techniques address the posterior mitral valve leaflet and include sliding leaflet plasty or chordal translocation [3]. Surgical approaches to the anterior mitral valve leaflet typically favor the creation of artificial chordae over leaflet resection [12]. The mitral valve annulus may require annular enlargement to reduce the area of the leaflets or plication to move the coaptation point and reduce the leaflet area [3]. Septal myectomy with a mitral-sparing approach is frequently performed to decrease the degree of LVOT obstruction if significant septal hypertrophy exists [17]. Although not the typical surgical approach to SAM in isolation, SAM was no longer an issue in both of our patients after cardiopulmonary bypass given that prosthetic valves were placed in the mitral position in the setting of concomitant significant mitral stenosis.

The development of a conduction disorder is a potential complication following open-heart surgery. In some cases, as seen in our first patient, severity may be severe enough to necessitate permanent pacemaker placement. The incidence of development of a conduction disorder is higher in multivalve surgeries when compared to isolated valve replacement. Other surgical risk factors that may increase the risk of postoperative permanent pacemaker requirement include prolonged cardiopulmonary bypass and aorta cross-clamping times [18].

## 4. Conclusion

The anesthetic management of these patients had compounding complexity due to the presence of multiple valvular pathologies as well as SAM resulting in dynamic LVOT obstruction. In addition to heightened vigilance during anesthetic induction and maintenance, TEE played a crucial role in monitoring and guiding both anesthetic and surgical management.

## Figures and Tables

**Table 1:** Kinetic mechanisms thought to cause systolic anterior motion of the mitral valve that may then cause left ventricular outflow tract obstruction [1, 10, 11].

Drag	Venturi
Dominant mechanism based on modern theory	Initially thought to be dominant mechanism
Ejection flow forces sweep MV leaflets toward the septum	Rapid velocities lift MV leaflets and/or suction leaflets into LVOT
Proportional to velocity through LVOT	Occurs at high velocities

Current theories based on detailed echocardiographic evaluation suggest that both mechanisms play a role in the development of SAM. MV: mitral valve; LVOT: left ventricular outflow tract; SAM: systolic anterior motion of the mitral valve.

**Table 2:** Comparison of hemodynamic goals of aortic stenosis, mitral stenosis, and systolic anterior motion of the mitral valve causing left ventricular outflow tract obstruction [13, 14].

Comparison of hemodynamic goals		
Aortic stenosis	SAM	Mitral stenosis
Rate: low-normal Avoid tachycardia	Rate: normal Avoid tachycardia	Rate: low-normal Avoid tachycardia
Rhythm: maintenance of NSR is especially important	Rhythm: maintenance of NSR	Rhythm: maintenance of NSR
Preload: maintain diastolic filling	Preload: increase	Preload: maintain or increase
Afterload: maintain or increase	Afterload: increase	Afterload: maintain
Contractility: maintain or increase Avoid depression	Contractility: decrease	Contractility: maintain

AS: aortic stenosis; MS: mitral stenosis; SAM: systolic anterior motion of the mitral valve.

## Data Availability

No data were used to support this study.
